# Tri©DB: an integrated platform of knowledgebase and reporting system for cancer precision medicine

**DOI:** 10.1186/s12967-023-04773-5

**Published:** 2023-12-06

**Authors:** Wei Jiang, Peng-Ying Wang, Qi Zhou, Qiu-Tong Lin, Yao Yao, Xun Huang, Xiaoming Tan, Shihui Yang, Weicai Ye, Yuedong Yang, Yun-Juan Bao

**Affiliations:** 1 State Key Laboratory of Biocatalysis and Enzyme Engineering, School of Life Sciences, Hubei University, Wuhan, 430062 China; 2 Wuxi Shengrui Bio-Pharmaceuticals Co., Ltd, Wuxi, 214174 Jiangsu China; 3 School of Computer Science and Engineering, Sun Yat-Sen University, Guangzhou, 510000 China; 4 Guangdong Province Key Laboratory of Computational Science, and National Engineering Laboratory for Big Data Analysis and Application, Sun Yat-Sen University, Guangzhou, 510000 China

**Keywords:** Cancer precision medicine, Knowledgebase, Reporting system

## Abstract

**Background:**

With the development of cancer precision medicine, a huge amount of high-dimensional cancer information has rapidly accumulated regarding gene alterations, diseases, therapeutic interventions and various annotations. The information is highly fragmented across multiple different sources, making it highly challenging to effectively utilize and exchange the information. Therefore, it is essential to create a resource platform containing well-aggregated, carefully mined, and easily accessible data for effective knowledge sharing.

**Methods:**

In this study, we have developed “Consensus Cancer Core” (Tri©DB), a new integrative cancer precision medicine knowledgebase and reporting system by mining and harmonizing multifaceted cancer data sources, and presenting them in a centralized platform with enhanced functionalities for accessibility, annotation and analysis.

**Results:**

The knowledgebase provides the currently most comprehensive information on cancer precision medicine covering more than 40 annotation entities, many of which are novel and have never been explored previously. Tri©DB offers several unique features: (i) harmonizing the cancer-related information from more than 30 data sources into one integrative platform for easy access; (ii) utilizing a variety of data analysis and graphical tools for enhanced user interaction with the high-dimensional data; (iii) containing a newly developed reporting system for automated annotation and therapy matching for external patient genomic data. Benchmark test indicated that Tri©DB is able to annotate 46% more treatments than two officially recognized resources, oncoKB and MCG. Tri©DB was further shown to have achieved 94.9% concordance with administered treatments in a real clinical trial.

**Conclusions:**

The novel features and rich functionalities of the new platform will facilitate full access to cancer precision medicine data in one single platform and accommodate the needs of a broad range of researchers not only in translational medicine, but also in basic biomedical research. We believe that it will help to promote knowledge sharing in cancer precision medicine. Tri©DB is freely available at www.biomeddb.org, and is hosted on a cutting-edge technology architecture supporting all major browsers and mobile handsets.

**Supplementary Information:**

The online version contains supplementary material available at 10.1186/s12967-023-04773-5.

## Introduction

Cancer is known to be a suite of complex diseases, usually driven by heterogeneous landscape of gene alterations entangled by environmental influences. The altered genes are involved in a multitude of interacting biological pathways or networks [1]. Current cancer treatments have been closely dependent on personal gene variation profile and the affected biological networks harboring the altered genes [2–5]. Thanks to the continuous advancement of cancer biology and rapid development of cancer therapeutic technologies coupled with the accessibility of large-scale genomic sequencing, cancer treatment has been at the forefront of the era of precision medicine [6, 7].

In the frame of precision medicine, strategies for cancer treatments have become highly precise, customized and diverse. On one hand, a particular gene variant might be present in different tumor microenvironments and could differentially respond to a specific therapeutic intervention, and vice versa, an individual disease could be related with multiple altered genes acting in different biological pathways and may fit for synergistic or sequential treatment strategies. On the other hand, the emergence of new therapies such as immunotherapy makes it possible to treat different cancers with the same agent given the cellular expression of specific biomarkers [8]. The plethora of knowledge and technologies regarding genetic profiles, disease settings, and therapeutic interventions have dramatically benefit patients, families, researchers, and clinicians.

However, it remains a challenging task to effectively present, utilize, and interpret that large amount of biologically interwoven information. Currently, the information is highly fragmented or restricted to commercial use, which in many cases hinders knowledge sharing, utilization, and interpretation for researchers. Therefore, it is essential to create a resource platform containing well-aggregated, carefully analyzed, optimally presented information for easy accessibility, accurate interpretation, and convenient annotation for the research community of cancer precision medicine.

Although several resources were developed in this regard, such as My Cancer Genome (MCG) [9], JAX Clinical Knowledgebase (JAX-CKB) [10], oncoKB [11], CIViC [12], Precision Medicine Knowledge Base (PMKB) [13], Cancer Genome Interpreter (CGI) [14], those resources suffer from limitations in that they only addressed some aspects of the cancer-related data and the data standards are diverse between them. For example, PMKB focused on variation interpretation, but not sufficiently addressed therapies related with the variations [13]. CGI aimed to annotate and interpret a wide range of cancer gene variants including those of unknown significance, but provided limited details on therapies and clinical studies [14]. OncoKB compiled information of biomarkers, tumor types, clinical interventions, and population sample annotations [11]. While oncoKB integrated contributions from clinical experts, such as physicians and oncologists, facilitating high clinical reliability, it does not provide rich annotation and interpretations, and hence the utility may tend to be restricted to clinical experts. Another notable source, CIViC, contains well-defined and carefully curated evidence records associated with individual or combination of genotype, disease and therapy [12]. MCG presents rich information regarding biomarkers, diseases, drugs, clinical studies, as well as text interpretations [15]. While CIViC and MCG share the commonality in completeness and complexity of the presented data, both of them lack mechanism information, recapitulative interpretations and detailed annotations, which are particularly needed for cancer biology researchers. Furthermore, most of those resources did not fully release their data publicly for free access or did not provide an automatic annotation system, making knowledge sharing even more difficult. That will prevent the utility of those sources among a broader scientific community and user population.

To address the limitations of existing resources, we have developed a new open-source platform “Consensus Cancer Core” or Tri©DB (www.biomeddb.org) that provides the most comprehensive information on cancer precision medicine with more than 40 annotation entities. By integrating and mining multifaceted information from more than 30 data sources, Tri©DB offers a variety of novel features on cancer precision medicine which are absent or incomplete in existing resources, such as real-time interactive analysis, manually curated recapitulative interpretations, rich graphical visualization, and automated annotation. We present them via a user-friendly web interface, thereby facilitating full access to the cancer-related data. We expect that our newly built platform accommodates the needs of a broad range of researchers and will promote the basic and translational research of cancer precision medicine.

## Materials and methods

### Data sources of the gene-disease-therapy triple-relationships

The gene-disease-therapy triple-relationships were referenced from multiple public sources, including Drug Approvals and Databases at Food and Drug Administration (drugs@FDA) (https://www.fda.gov/drugs/) and National Comprehensive Cancer Network Clinical Practice Guidelines In Oncology (NCCN Guidelines®) (https://www.nccn.org). We extracted the drug indication information from FDA drug label files and NCCN guideline files by manual reading to obtain the gene-disease-therapy triple relationships. The PubMed API tool “E-utilities” was used for automatic searching and retrieval of the literatures containing the keyword combination “gene + cancer + therapy” in the title or abstract. The most recent relevant literatures were shown.

### Genetic data processing and analysis

The baseline gene annotations, such as “Gene Alias”, “Entrez_geneID”, “HGNC_ID”, “Ensemble_ID”, and “RefSeq Transcript” were obtained using the R package “BioMart” and “org.Hs.eg.db” (GRCh37). The functional annotations of gene variants, such as amino acid change and variant type were performed using ANNOVAR [16]. The gene names and gene fusions were normalized across different sources and standardized based on the HGNC nomenclature [17, 18]. The gene variants were normalized and converted to the HGVS format (protein level) [19]. References to genes or gene variants were provided by linking to external sources, such as GENECARDS [20], dbSNP [21], COSMIC Cancer Mutation Census [22], ClinVar [23], OMIM [24], and COSMIC Cancer Gene Census (CGC) [25].

The level of clinical significance of gene variants was categorized into five classes, i.e., “Pathogenic”, “Likely Pathogenic”, “Likely Benign”, “Benign”, “Uncertain” using three resources, i.e., COSMIC, ClinVar, and VIC [22, 23, 26].

The population carrier rate of somatic variants was calculated using the datasets of AACR Project Genomics Evidence Neoplasia Information Exchange (GENIE) cohort [27]. The population carrier rate of Chinese somatic variants was compiled from the study by Zhang, et al. [28]. The population carrier rate of germline variants was based on The Cancer Genome Atlas (TCGA) cohort analysed by Huang, et al. [29]. The population carrier rate was calculated and presented on the gene level and cancer-type level, respectively. On the gene level, the population carrier rate of each variant on a specific gene among the whole cohort was visualized in a lollipop-style graph. The population carrier rate on the gene level was visualized in a lollipop-style graph, within which the protein domain architectures of the genes were annotated via Pfam database API [30]. On the cancer level, the population carrier rate of all variants among a specific cancer cohort was visualized in a bar chart format using the JavaScript visualization tool ECharts [31]. For performance optimization, the calculation was only performed at runtime when the page was requested by users from the web interface.

The pathways related to each gene were displayed using DiagramJS widget of the REACTOME database and API of the KEGG database [32, 33]. The interaction network describing the interaction partners of each gene was implemented via API from the Network of Cancer Genes (NCG) [34].

### Therapy data sources and processing

The drug attributes, such as “Drug Name”, “Drug Brand”, “Approval Time”, “Mechanism of Action” and “Dosage” were mainly extracted from drug@FDA. The drug names from different sources were standardized and normalized based on United States Adopted Names (USAN) and DrugBank [35]. The therapies were classified into single-target inhibitors, multi-target inhibitors, monoclonal antibodies, bi-specific antibodies, combination, immunotherapies, and cell therapies based on the molecular properties and mechanism of action indicated in the attribute “Drug Type”.

The clinical trials related to specific therapies and indications were compiled from the datasets from https://clinicaltrials.gov. All records of clinical trials were downloaded in the XML format as of April 24, 2021. The matched records were also linked to the corresponding webpages at https://clinicaltrials.gov via API. All matching results were further manually confirmed.

The interactive viewer for the three-dimensional conformer of small molecular drugs was implemented via the PubChem Widgets [36] and that for the three-dimensional structure of antibody drugs via RCSB PDB (www.rcsb.org) structural view plug-in library pdbe-molstar [37].

### Disease data processing

Cancer type names are highly mosaic among different data sources. Two disease ontology resources, i.e., NCI thesaurus (NCIt, https://ncithesaurus.nci.nih.gov) and OncoTree [38] (https://oncotree.mskcc.org) were used for cancer name normalization. The cancer type names were standardized using the OncoTree ontology and additional links to NCIt classification were also provided. To enhance searchability and accessibility of the cancer types with multiple synonyms, the aliases of cancer types were compiled based on the NCIt ontology and enabled to be searched.

The cancer-specific pathway graphs were obtained through literature review by searching PubMed website (www.pubmed.ncbi.nlm.nih.gov) using the keyword combination “cancer + pathway” or “cancer + mechanism”. The literatures were manually read to select the most relevant based on four criteria in descending priority: (1) The paper is a review article; (2) The paper has a higher citation than others; (3) The pathway in the paper was constructed based on experimental evidences with corresponding citations; (4) The experimental evidences include molecular biology experiments and animal model pathology experiments. The searching for the pathway literatures covered the time period Jan. 2006-Aug. 2022. The hyperlinks for the selected references were provided.

### Construction of gene-disease-therapy interconnecting network

The interconnecting networks of the gene-disease-therapy triple-relationships were visualized using the JavaScript graph library Cytoscape.js [39]. The disease-gene/gene-therapy dual relationships were represented as edges, and individual genes, diseases and therapies represented as nodes. The sizes of gene nodes and the weights of disease-gene edges are proportional to the accumulated carrier rate of gene-level alterations in cancer-specific cohort of GENIE [27]. For each node, multiple external references can be directed to, such as MedlinePlus (https://medlineplus.gov/) and Therapeutic target DB [40] for therapies, Uniprot [41] and GeneCards [20] for genes, MalaCards [42] for diseases.

### Biological and clinical interpretations

The recapitulative text interpretations “Functional and Clinical Implications” and “Clinical Interpretations” were prepared through intensive literature reviews, manual curation, and detailed summary. Multiple sources were searched to obtain the relevant information, *i.e.*, from the PubMed database using the keyword combination “gene + cancer” to extract gene functions and cancer causal mechanisms, from the FDA drug database (https://www.fda.gov/drugs/) and NCCN Guideline® (https://www.nccn.org) to extract the approval information or community consensus, from the clinical trial database (www.clinicaltrials.gov) to extract the clinical trials related to the therapies. The information was summarized and compiled to our own interpretations. Each record of the interpretations was reviewed by at least one expert in translational precision oncology.

### Web server implementation

The web server was developed in a MVVM (Model-View-ViewModel) framework in the .Net core environment which supports cross-platform application. All data was managed with the MySQL database system. Tri©DB is maintained on a Linux-based Apache web server and runs in a Docker container. The database supports most of the mainstream web browsers, such as Chrome, Firefox, Microsoft Edge, and Safari and various mobile handsets.

### Annotation and reporting system

The annotation and reporting system comprises a series of open-source R/Python packages, including SigMiner [41] (version 2.1.9), Maftools [43] (2.14.0), NMF [44] (version 0.25), SigProfilerMatrixGenerator [45] (version 1.2.13), and SigProfilerPlotting [45] (version 1.3.6).

### Data visualization

Multiple visualization tools or modules were used for data visualization, including the JavaScript graphing tools ECharts (https://echarts.apache.org, version 4.0, 2020), Highcharts JS (https://www.highcharts.com, version 9.2.2, 2021), Cytoscape.js (https://js.cytoscape.org, version 4.0, 2020), g3lollipop.js (https://github.com/G3viz/g3lollipop.js, version 0.5.0, 2019), and various web APIs.

## Results

### Overview of the architecture and main contents of Tri©DB

**Fig. 1:**
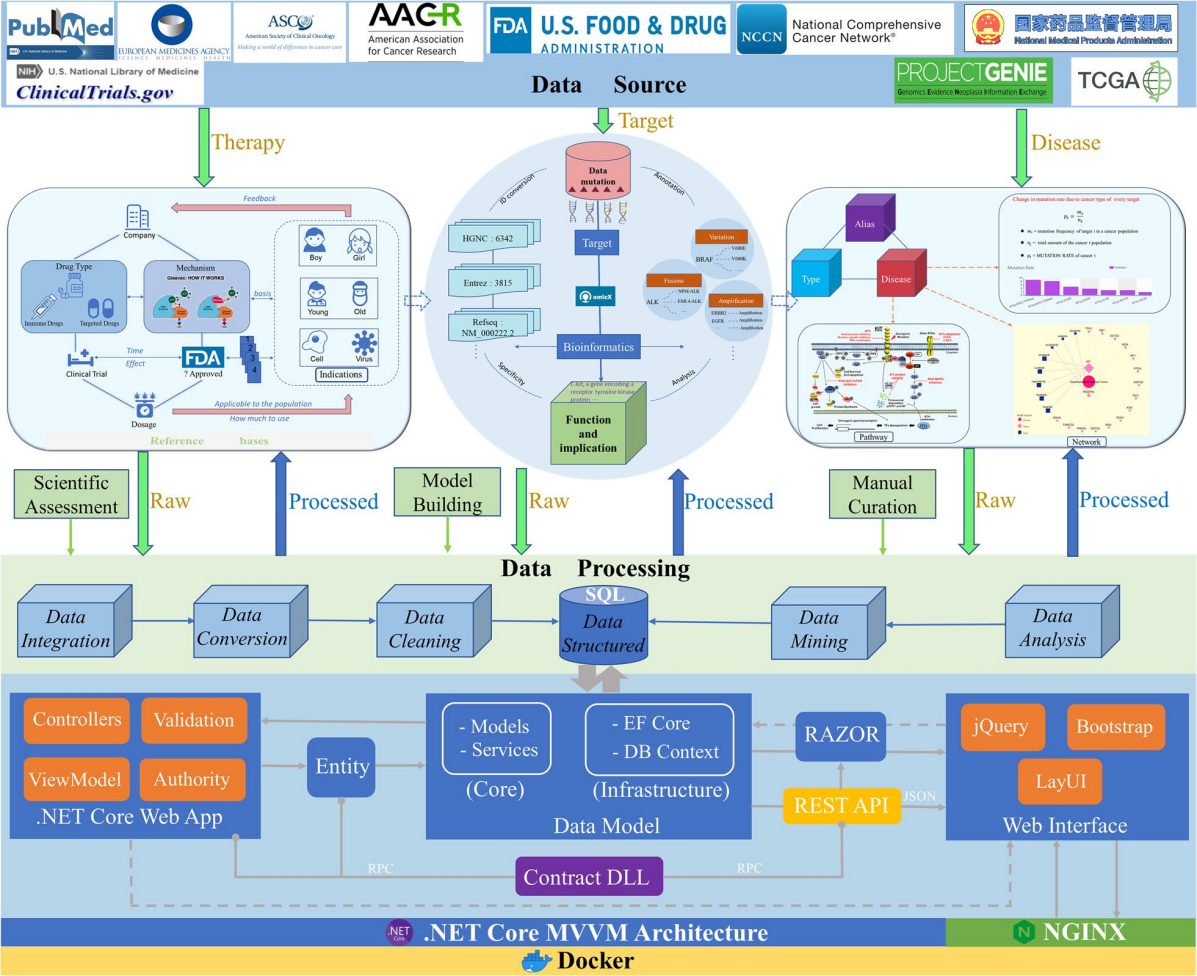
An overview of the flowchart of data construction of Tri©DB and architecture of the platform. Top: illustration of database elements and contents; Middle: key data processing procedures; Bottom: the layout and key techniques in the architecture of Tri©DB

The data was mined and harmonized from more than 30 sources and the data architecture was designed in the advanced MVVM framework with separated layers for data access on multiple levels. A reporting system was also developed in the backend for automated annotation of external variant data, enabling scalable and portable implementation of patient data interpretation (Fig. 1).

**Fig. 2:**
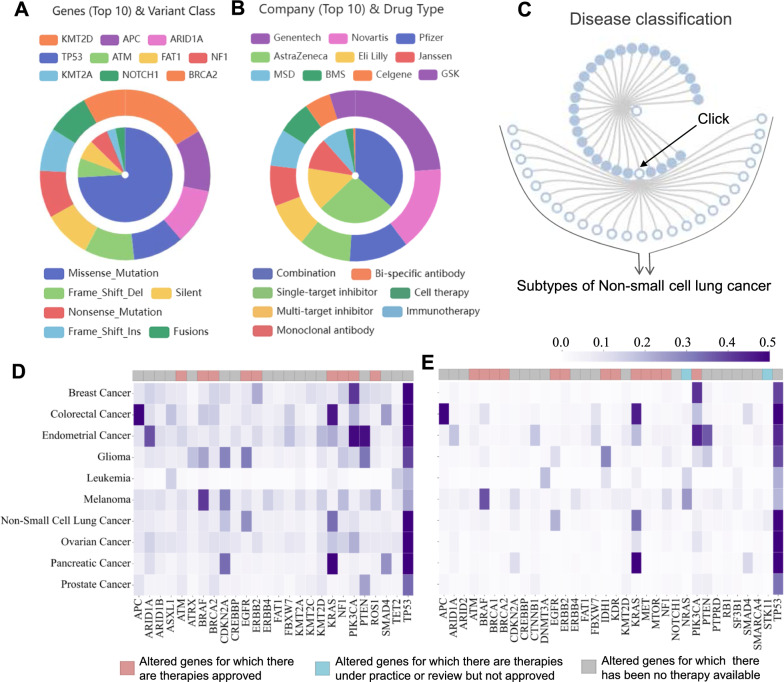
Graphical presentation of the key statistics of data included in Tri©DB. **A** The gene mutation classifications (inner circle) and the top genes with the most mutated loci (outer circle). **B** The drug classifications (inner circle) and top manufactures (outer circle). **C** The hierarchical classification of the cancer types mentioned in Tri©DB. Each dot can be clicked to show the subtypes of each main cancer type. **D** Heatmap presentation of the prevalence of gene alterations in the major cancer types listed in Tri©DB based on the GENIE cohort. **E** The same as that in **C** but only for alterations annotated as pathogenic/likely pathogenic. Only the most commonly altered or well-known cancer driver genes are shown. The existence of drugs for the altered genes is indicated in coloured bars on top of the heatmap

The database comprises 398,180 population-level alterations on 1,308 altered genes, 232 actionable genotypes, 84 cancer types, 268 therapies linked by 948 associations, 1,847 clinical trials, and 40 annotation entities by mining more than 33 external databases and numerous literatures (see “Methods and Materials” section). Key statistic summary of genes, diseases, and therapies is presented in Fig. 2. It is found that the majority of the alterations are missense mutations, and KMT2D contains the most mutated loci, followed by APC, ARID1A, and TP53 (Fig. 2A). Up to now, combination products are the major form of cancer therapies and Genentech developed the most new molecular drugs, followed by Novartis and Pfizer (Fig. 2B). For the cancer types mentioned in Tri©DB, a clickable circle tree was used to demonstrate their classification in a hierarchical structure (Fig. 2C). We further assessed the profile of gene mutation prevalence in the major cancer types (Fig. 2D and E). Several of the hotspot genes have been successful targets for the development of anticancer drugs, such as BRAF, EGFR, and KDR, while the majority of commonly altered genes have no drugs available.

### Derivation of data in gene-, disease-, and therapy-oriented tabular format in the first layer of data

The content of the knowledgebase can be accessed in two layers from the web interface to accommodate the needs of diverse user groups. The first layer provides all gene-disease-therapy triple-relationships along with fifteen annotation attributes in a brief tabular format in the gene-, disease-, and therapy-oriented view. The second layer presents the detailed annotation and interpretation in a separate page for each entity.

The tabular data presentation in the first layer aims to provide an overview yet with sufficient annotation of the gene-disease-therapy triple-relationships. The data can be obtained in the gene-, disease-, therapy-oriented view separately. The three oriented views contain various annotation information (Table 1).

**Table 1:** The annotatiaon attributes in the gene-, disease-, and therapy-oriented tabular views

Attributes	Gene	Disease	Therapy
Gene	+	+	+
Alteration	+	+	+
Negative genotype	+	+	+
Disease	+	+	+
Drug name	+	+	+
Direct target	+	+	+
Specificity	+	+	+
Evidence level	+	+	+
Resistance	+		
Clinical significance (ClinVar)	+		
Clinical significance (COSMIC)	+		
Clinical significance (VIC)	+		
Variant classification	+		
Carrier rate (GENIE)		+	
Carrier rate (Chinese)		+	
Carrier rate (Germline)		+	
Drug type			+
Drug brand			+
Approval time			+

The implementation of the three separate views was done by recognizing the complexity of the relationship between gene alterations, disease phenotypes and therapies, where an individual alteration might occur in different disease contexts and are predictive of responses to distinct therapeutic interventions, and vice versa, an individual disease could be related with multiple gene alterations involved in different biological pathways and call for differentiated therapy protocols. The design will facilitate rapid access to key information for users from diverse background without digging into additional details, and the data can be seamlessly integrated into third-party reporting systems or annotation pipelines.

### Unique features provided by the first layer of data

In the first layer of the data, our database provides two unique features, which have not been present in other similar resources. Firstly, in addition to the regular positive genotypes (gene + alteration), we added the attribute “Negative Genotypes” to indicate the opposing genotypes, which may not respond to a specified therapy or is associated with poor prognosis. For example, the recent anti-EGFR therapies for metastatic colorectal carcinoma should not be used in patients with KRAS mutations [46].

Secondly, we identified “Direct Target” in the therapy-oriented tabular view for each therapy for which the matching genotypes are different from the directly targeted genes. A notable example is the therapies for KRAS mutated carcinomas. Before the successful development of KRAS inhibitor Sotorasib, the therapeutic studies for KRAS-mutant cancers focused on targeting downstream effectors in the RAS-RAF-MEK-ERK pathway, such as the MEK inhibitor Trametinib in combination with chemotherapy for patients with metastatic non-small cell lung cancer (NSCLC) [47].

### Derivation of detailed annotation information for each gene-disease-therapy triple-relation in the second layer of data

The second layer of data in Tri©DB aims to provide detailed annotation for each record of gene-disease-therapy triple-relationships, offering a rich breath of cancer-related knowledge in structured attributes, such as functional annotations, interpretations, population carrier rate, and interactive networks.

**Fig. 3:**
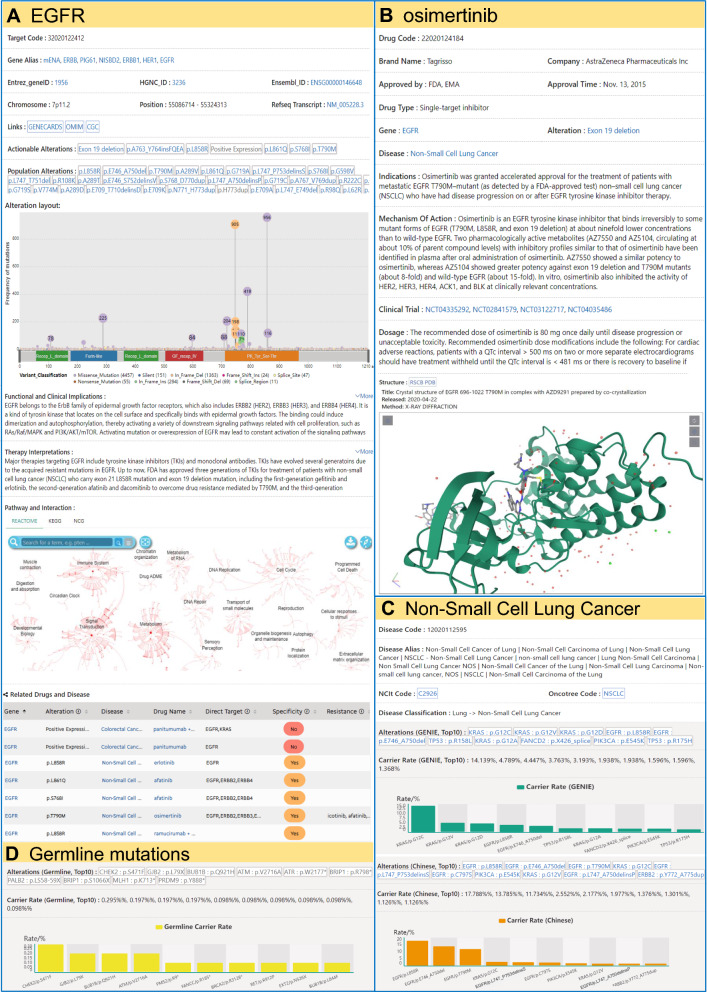
An example of the detailed report for the gene EGFR, therapeutic drug osimertinib, and disease NSCLC in the second layer of data in Tri©DB. **A** Details of EGFR, including basic genomic annotation, population mutation profile, functional interpretation, therapy interpretation, pathway and interaction. **B** Details of the drug osimertinib, including approval information, indication, mechanism of action, clinical trial, and the three-dimensional structure. **C** Details of NSCLC, including disease classification, mutation carrier rate in the Western and Chinese population. **D** Germline mutation carrier rate was also provided in the disease report

As an example, the details for the gene EGFR (epidermal growth factor receptor), therapeutic drug osimertinib, and disease NSCLC was illustrated in Fig. 3. For the gene EGFR, the results show that 98 different alterations occurring among the GENIE cancer cohort, whereas the most common alterations are exon 21 missense mutations, exon 19 deletion mutations, and exon 20 mutation T790M (the lollipop graph in Fig. 3A). To provide mechanistic explanations for the pathogenesis of the genes and the therapies relevant to the specific gene, the recapitulative interpretations for each gene, *i.e.*, “Functional and Clinical Implications” and “Clinical Interpretations” were constructed based on intensive literature review and manual curation. In complement with the text interpretation, a graphical presentation “Pathway and Interaction” was provided by connecting to three external resources, *i.e.*, REACOME [33], KEGG [32] and NCG [34].

The small molecular inhibitor osimertinib is the third-generation TKI to overcome resistance mediated by EGFR mutations including T790M. The report for this drug presents multi-dimensional information on the mechanistic and clinical level, such as the three-dimensional complex structure of osimertinib and EGFR-T790M, and the clinical trials for validating efficacies of osimertinib (Fig. 3B). This information might be of particular interest for health care professionals or patient groups who are seeking to enroll in trials relevant to a specific drug.

The cancer-type level calculation of the population carrier rate of gene alterations among the NSCLC cohort shows that KRAS mutations are the most common somatic alterations in the Western population of the NSCLC (> 20%), while EGFR variations account for the largest proportion in the Chinese population (Fig. 3C). The results of the population carrier rate for the cancer cohort provide a brief idea of the fraction of patient population who may benefit from the therapies targeting a specific gene.

### Unique features of the detailed annotation information in the second layer of data

Tri©DB provides multiple unique features for the detailed annotation in the second layer of data. The most notable includes the following three. First, Tri©DB collected and compiled the cancer-level population carrier rate for germline mutations (Fig. 3D). This information was generally ignored by other resources probably due to the overall low population prevalence [25]. For example, the germline mutations are rare in NSCLC with the most common mutation occurring in the homologous recombination repair (HRR) gene CHEK2 (S471F, 0.295%) (Fig. 3D). The top germline mutations were also found in several other HRR genes, such as ATM (V2716A, 0.197%), FANCC (R185*, 0.098%), BRCA2 (R3128*, 0.098%). (Fig. 3D).

**Fig. 4:**
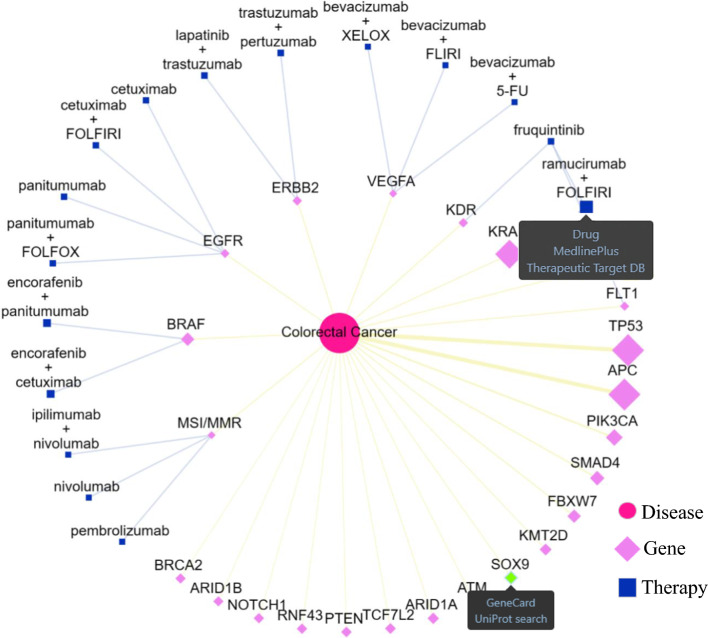
Notable features of the detailed annotation information for the example disease colorectal cancer. The interactive network presentation of the gene-disease-therapy triple-relationship with the colorectal cancer as the network centre. The sizes of gene nodes and the weights of disease-gene edges are proportional to the accumulated carrier rate of gene-level alterations in cancer-specific population cohort. Each node of genes or therapies is linked to multiple external resources

Secondly, Tri©DB constructed the disease-gene-therapy triple-relationships in a disease-centred manner and dynamically generated interactive networks for the triple-relations (Fig. 4). The network presentation will help to elucidate the genetic and therapeutic landscape for a specific cancer type. Users can interact with the networks by refining the layout or redirecting to internal and external resources for further details of each node in the network. An example of the landscape for the colorectal cancer is demonstrated in Fig. 4. It shows that more than 10 altered genotypes, such as TP53, APC, KRAS, and BRAF, and the global DNA instability (*i.e.*, Microsatellite Instability High or Mismatch repair deficiency, namely MSI-H/dMMR) have been found to be associated with colorectal cancer. Nine of them have approved therapies to act on their altered form, such as BRAF, ERBB2, KRAS, EGFR, VEGFA, VEGFR/KDR, FLT1, FLT4, and MSI/MMR.

Thirdly, our database presents the mechanism-based cancer-specific pathways, which have been largely scattered around literatures or databases. We collected and mined those pathways by thorough literature survey. The links of the source of origin are also provided. An example is the pathway map for colorectal cancer [48]. It is shown that colorectal cancer can develop via multiple genetic (APC, KRAS, TP53, BRAF, MMR) and epigenetic (MLH1) factors involving several distinct but intertwined pathways, such as Wnt signalling pathway, Myc signalling pathway, MAPK pathway, TGF-β pathway, and serrated neoplasia pathway. The mechanism pathways of cancer types in combination with the disease-gene-therapy networks provide valuable pivot points for elucidating the pathogenic and therapeutic landscape of specific cancers.

### Automatic annotation and generation of the portable interpretation report

In addition to the interactive access to the data in Tri©DB, our open-source platform also contains web interface reporting system facilitating automated annotation and interpretation of user-provided bulk variant data.

**Fig. 5:**
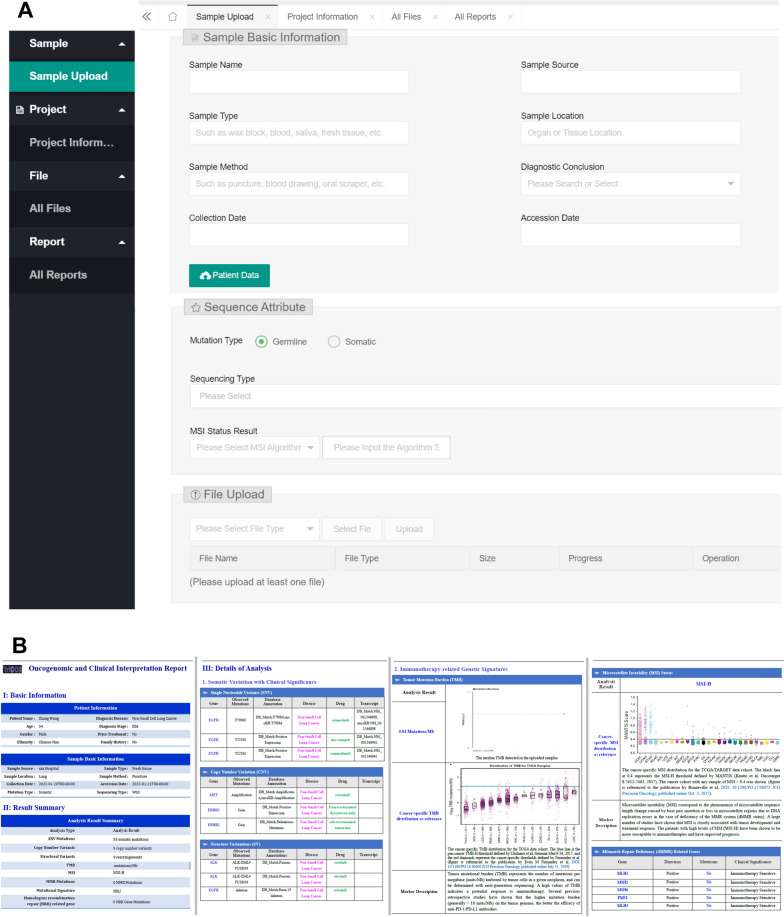
Overview of the reporting system. **A** The web interface of the file uploading module allowing users to provide various mutation types and sequencing modalities. **B** Preview of the integrated report for user-input variant data

The reporting system supports a variety of variant classes (including SNV, CNV, SV, MSI, somatic mutations, and germline mutations) for uploading in standard or software-specific formats. The system also allows users to designate the mutation types (i.e., somatic or germline) and sequencing modalities (i.e., WGS, WES, or Gene Panel) for adapting to distinct analysis workflow or knowledgebase contents (Fig. 5A). Considering the diverse types of genomic alterations relevant to cancer, the annotation system at first performs multiple analysis, such as tumor mutation burden (TMB) calculation, MMR gene detection, HRR gene detection, mutational signature identification, and subsequently matches each variant signature against Tri©DB for extracting multiple annotations, such as gene functions relevant to cancer, clinical trials, mechanism of actions, drug resistance, and et al. Finally, all the analysis and annotation results are organized and integrated in a single report file enabling easy dissemination and communication among researchers (Fig. 5B and Additional file 1).

### Performance evaluation of Tri©DB in variant annotation

To evaluate the performance of Tri©DB in variant annotation, we made therapy matching using Tri©DB in two scenarios, i.e., an individual patient sample and a patient cohort and compared the matching with that from other resources.

Firstly, an artificial individual patient was created to harbor 37 variants from 10 genes representing a wide range of variant types (SNV, gene amplification, gene loss, and fusion) and driver gene categories (such as cell proliferation, apoptosis inhibition, angiogenesis, DNA repair, and genomic instability) (Additional file 2). The variants were annotated using Tri©DB and compared the annotation with that using two notable knowledgebases of similar kind, *i.e.* oncoKB which was recognized by FDA to support cancer precision medicine practices and MCG (My Cancer Genome) which was commercially licensed. Based on FDA evidences and clinical guidelines, a total of 420 treatment options for the 37 variants (i.e. variant-disease-therapy triples) were annotated by Tri©DB sharing 97.9% of those by oncoKB and 99.6% of those by MCG. Tri©DB annotated 133 more treatments than oncoKB and 137 than MCG, accounting for 46% of the shared list (Additional file 2). The additional treatments annotated by Tri©DB are mainly targeted antibody drugs or immunotherapy drugs, such as necitumumab and durvalumab for EGFR, and pertuzumab for ERBB2.

We then made therapy matching for a patient cohort from a prospective clinical trial called I-PREDICT dedicated to investigate individualized cancer therapy (NCT02534675) [49]. This clinical trial administered individualized therapies for 83 patients diagnosed of a wide range of cancer types and has been used for therapy recommendations by MOAlmanac, an integrative platform of clinical interpretation [50]. A total of 524 gene variants from the 83 patients were extracted and therapy matching was made by Tri©DB on the per-variant per-patient basis. Based on FDA evidences or clinical guidelines, 59 variant-patient pairs were administered with therapies in the clinical trials, 56 of which (94.9%) are overlapped by Tri©DB involving 31 patients (Additional file 3). The three annotations missed by Tri©DB are all tamoxifen, which was approved by FDA 40 years ago. The overlap proportion is significantly higher than that for MOAlmanac (20 variant-patient pairs, 33.8%), probably because MOAlmanac focused on providing best therapy recommendations but not a comprehensive list based on the global molecular profile of each patient.

The comparison results highlight the high completeness and accuracy of the annotations by Tri©DB. The high consistency of Tri©DB with the clinical trial practices supports its utility in clinical applications.

## Discussion

The last several decades have witnessed the identification of complex molecular mechanisms of tumorigenesis and discovery of ever-growing genomic alterations related to cancers. Accordingly, the therapeutic interventions against the alterations in cancers have also rapidly advanced and accumulated. For example, cell therapies, which introduce engineered or functional cells into patients to fight cancers, have shown rapid growth in recent years in clinical investigations or pharmaceutical markets with notable examples including CAR-T, TCR-T, and CAR-NK [51, 52]. It was estimated that there have been more than 2700 active cell therapy agents in clinical or preclinical development and the tested targets of the therapy agents have expanded from several frequently used genes, *i.e.*, CD19, BCMA, CD22, CD20 to more than 50 genes [53]. Another type of emerging cancer immunotherapy, *i.e.*, immune checkpoint inhibitors (ICIs), which block the immune checkpoint molecules and reactivate immune response against cancer cells, experienced even more dramatic growth since the FDA approval of the PD-1/PD-L1 inhibitors pembrolizumab and nivolumab for treatment of melanoma in 2014 [54]. ICIs are usually used in combination with other therapies, especially targeted therapies. To date, nearly 300 targets or target combinations are being investigated in ICIs-related therapy regimens and more than 4000 clinical trials involving the targets are being conducted globally [55]. Recently, the nanomaterial-based delivery system has been gaining attentios due to its potential in overcome the limitations of cell therapies or immunotherapies in controlling release of the therapeutic agents or minimizing the off-target toxicities [56, 57]. A plenty of nanomaterials have been developed for drug delivery, such as liposomes, dendrons, micelles, metal nanoparticles, and even plant-derived nanovesicles, although most of them have not reached clinical stages or marketplaces [58, 59].

Therefore, this rapidly accumulated information forms a multi-dimensional complicated knowledge network, making it challenging to effectively utilize the information even for professionals.

In the current study, we aim to provide a highly confident open-source platform that delivers not only the tables or forms, but also interactive analysis, various unique contents, and automated reporting system, thereby accommodating the needs of a broad range of researchers, who study cancer genetics, tumorigenesis, drug development, and even clinical investigations. The unique features provided by Tri©DB are absent or incomplete in other similar resources, and particularly, the automatic annotation system has been lacking in academic settings, making our knowledgebase a major advance and a valuable alternative to current similar resources (Table 2). The features are exemplified below.

I. The narrative paragraphs of interpretations, such as “Functional and Clinical Implications” and “Clinical Interpretations” will serve as primers to enhance knowledge sharing among communities of oncologists, pathologists, and clinical experts. They were manually curated by group members and carefully examined by experts in translational precision oncology. To the best of our knowledge, Tri©DB is the first such resource that provides manually curated narrative interpretations for cancer precision medicine data.
II. The construction of interactive network graphs for elucidating the complex gene-disease-therapy triple-relationships will allow oncological professionals to quickly obtain the genetic and pharmacogenomics landscape of specific cancers and get insights on drug development roadmaps.
III. A multitude of unique contents in the detailed annotation pages were offered in Tri©DB, such as germline mutations, population carrier rate of Chinese population, immunotherapies, and cell therapies, which were developed rapidly but have been generally ignored by current resources of similar kind.
IV. Various interactive analysis and graphic visualization tools were used to analyse and present the high-dimensional data structures in Tri©DB, such as lollipop graphs, network graphs, bar charts, pie charts, and hierarchical trees, facilitating enhanced access and interpretability of the complex cancer precision medicine data.

**Table 2:** Comparison of key features of Tri©DB with those of other similar resources

Database	Release time	Key knowledge domain	Primary presentation	Graphical presentation	Meta data	Pathway/interaction	Germline mutations	Immuno therapy	Cell therapy	Data access	Annotation report
oncoKB	2017	Gene alterations, Therapies, Diseases, Brief interpretations	Tables, Texts	Lollipop graphs, Barcharts	Functional annotations, Population frequencies, Functional domains, Drug resistance	No	No	Yes	No	Browse, Search, API	Yes, license needed
CIViC	2017	Gene alterations, Therapies, Diseases, Clinical trials, Assertive interpretations	Tables, Texts	/	Functional annotations, Population frequencies, Drug resistance, Literature references	No	No	Yes	No	Browse, Search, API, Download	No
MCG	2011	Gene alterations, Therapies, Diseases, Clinical trials, Brief interpretations	Forms, Texts	Barchats, Pathway maps	Functional annotations, Population frequencies, Literature references	Yes	No	Yes	No	Browse, Search	No
PMKB	2017	Gene alterations, Therapies, Diseases, Interpretations	Forms, Texts	/	Functional annotations, Drug resistance, External cross-links	No	No	No	No	Browse, Search, API	No
CGI	2018	Gene alterations, Therapies, Diseases	Tables, Texts	/	Functional annotations, Drug resistance, External cross-links	No	No	No	No	Annotate, Filter, API, Download	No
JAX-CKB	2016	Gene alterations, Therapies, Diseases, Clinical trials, Interpretations	Tables, Forms, Texts	/	Functional annotations, Drug resistance, External cross-links, Literature references	No	Not explicit	Yes	No	Partial access, license needed	No
Tri©DB	2022	Gene alterations, Therapies, Diseases, Clinical trials, Recapitulative interpretations	Tables, Forms, Texts	Pathway maps, Interaction networks, Lollipop graphs, Barcharts	Functional annotations, Population frequencies, Functional domains, Drug resistance, Molecular structures, External cross-links, Literature references	Yes	Yes	Yes	Yes	Browse, Search, API, Download	Yes

We believe that the multifaceted information will promote the basic and translational research of cancer precision medicine and provide support for data-driven clinical decision.

### Supplementary Information

**Additional file 1**: Supplementary_File_1_report.pdf, A demo report generated by the automated annotation system of Tri©DB.

**Additional file 2**: Supplementary_File_2.xlsx, Performance evaluation of annotation by Tri©DB by comparison with oncoKB and MCG.

**Additional file 3**: Supplementary_File_3.xlsx, Performance evaluation of annotation by Tri©DB via therapy matching for a cohort from the clinical trial I-PREDICT.

## Data Availability

The database is freely available at www.biomeddb.org for academic users by downloading, searching, and API. The datasets supporting the conclusions of this article are included within the article and its additional files. We make all efforts to promote the sharing of data based on community-recognized standards to fulfill the FAIR principles.
