# Supplementary material for: Tri©DB: an integrated platform of knowledgebase and reporting system for cancer precision medicine
Source: J Transl Med. 2023 Dec 6;21:885. doi: 10.1186/s12967-023-04773-5 (PMC10702018; doi:10.1186/s12967-023-04773-5)
Supplement: Supplementary file 1 — Additional file 1: Supplementary_File_1_report.pdf, A demo report generated by the automated annotation system of Tri©DB. [file 12967_2023_4773_MOESM1_ESM.pdf]

Oncogenomic and Clinical Interpretation Report

I: Basic Information

Patient Information			
**Patient Name :**	Zhang Wang	**Diagnosis Disease:**	Non-Small Cell Lung Cancer
**Age :**	54	**Diagnosis Stage :**	IIIA
**Gender :**	Male	**Prior Treatment :**	No
**Ethnicity :**	Chinese Han	**Family History :**	No

Sample Basic Information			
**Sample Source :**	xxx Hospital	**Sample Type :**	Fresh tissue
**Sample Location :**	Lung	**Sample Method :**	Puncture
**Collection Date :**	2023-01-20T00:00:00	**Accession Date :**	2023-01-21T00:00:00
**Mutation Type :**	Somatic	**Sequencing Type :**	WGS

II: Result Summary

Analysis Result Summary	
**Analysis Type**	**Analysis Result**
**SNV Mutations**	35 somatic mutations
**Copy Number Variants**	9 copy number variants
**Structural Variants**	9 rearrangements
**TMB**	mutations/Mb
**MSI**	MSI-H
**MMR Mutations**	0 MMR Mutations
**Mutational Signature**	SBS/
**Homologous recombination repair (HRR)-related gene**	0 HRR Gene Mutations

III: Details of Analysis

1. Somatic Variation with Clinical Significance

Single Nucleotide Variants (SNV)

Gene	Observed Mutations	Database Annotation	Disease	Drug	Transcript
EGFR	T790M	DB_Match:T790M,oncoKB:T790M	Non-Small Cell Lung Cancer	osimertinib	DB_Match:NM_001346898, oncoKB:NM_001346898
EGFR	T523M	DB_Match:Positive Expression	Non-Small Cell Lung Cancer	durvalumab	DB_Match:NM_001346941
EGFR	T523M	DB_Match:Positive Expression	Non-Small Cell Lung Cancer	sugemalimab	DB_Match:NM_001346941

Copy Number Variation (CNV)

Gene	Observed Mutations	Database Annotation	Disease	Drug	Transcript
MET	Amplification	DB_Match:Amplification,oncoKB:Amplification	Non-Small Cell Lung Cancer	crizotinib	
ERBB2	Gain	DB_Match:Positive Expression	Non-Small Cell Lung Cancer	Fam-trastuzumab deruxtecan-nxki	
ERBB2	Gain	DB_Match:Deleterious Mutations	Non-Small Cell Lung Cancer	ado-trastuzumab emtansine	

Structure Variantions (SV)

Gene	Observed Mutations	Database Annotations	Disease	Drug	Transcript
ALK	ALK-EML4 FUSION	DB_Match:Fusions	Non-Small Cell Lung Cancer	ceritinib	
ALK	ALK-EML4 FUSION	DB_Match:Fusions	Non-Small Cell Lung Cancer	alectinib	
EGFR	deletion	DB_Match:Exon 19 deletion	Non-Small Cell Lung Cancer	erlotinib	

2. Immunotherapy-related Genetic Signatures

Tumor Mutation Burden (TMB)

**Analysis Result**	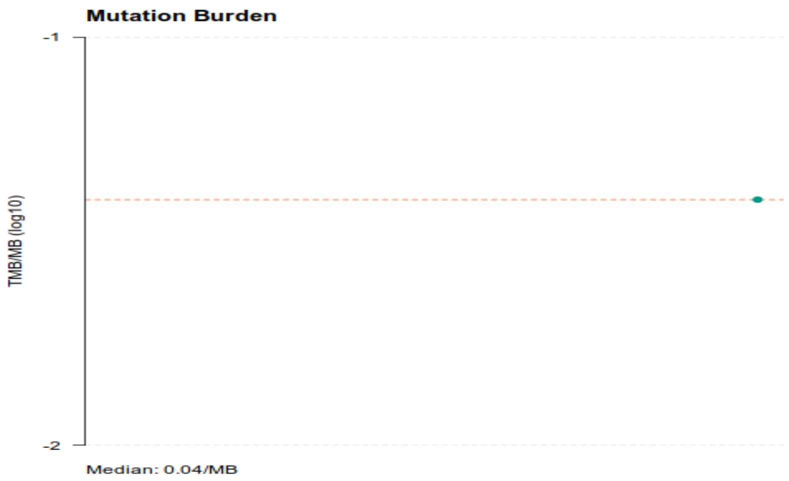
**0.04 Mutations/Mb**	The median TMB detected in the uploaded samples
**Cancer-specific TMB distribution as reference**	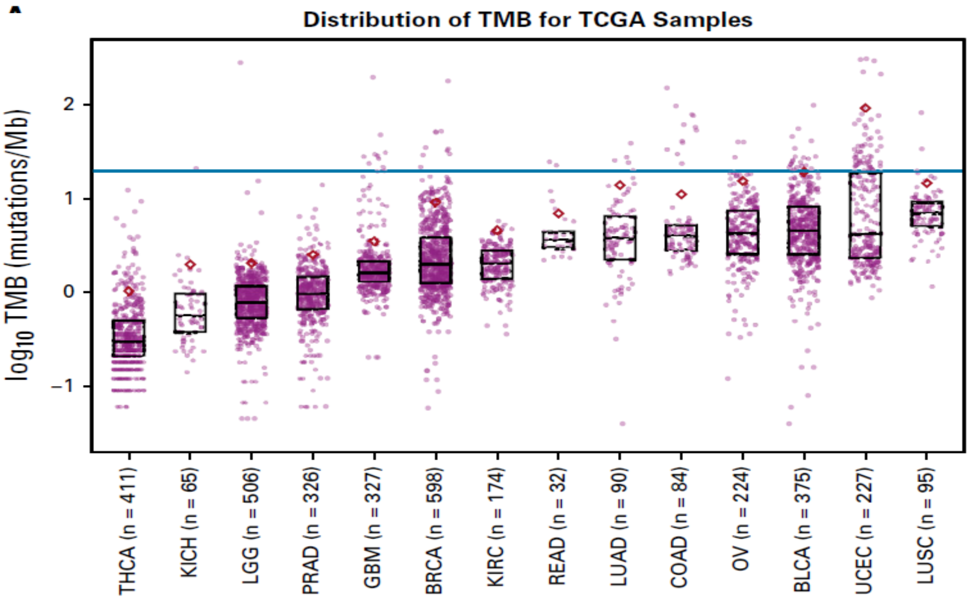 The cancer-specific TMB distribution for the TCGA data cohort. The blue line is the pan-cancer TMB-H threshold defined by Chalmers et al, Genome Med 9:34, 2017, and the red diamonds represent the cancer-specific thresholds defined by Fernandez et al. (figure is referenced to the publication by Evan M Fernandez et al, DOI: 10.1200/PO.18.00400 JCO Precision Oncology, published online July 31, 2019)
**Marker Description**	Tumor mutational burden (TMB) represents the number of mutations per megabase (muts/Mb) harbored by tumor cells in a given neoplasm, and can be determined with next-generation sequencing. A high values of TMB indicates a potential response to immunotherapy. Several previous retrospective studies have shown that the higher mutation burden (generally > 10 muts/Mb) on the tumor genome, the better the efficacy of anti-PD-1/PD-L1 antibodies.

Microsatellite Instability (MSI) Status

Analysis Result	**MSI-H**

Cancer-specific MSI distribution as reference

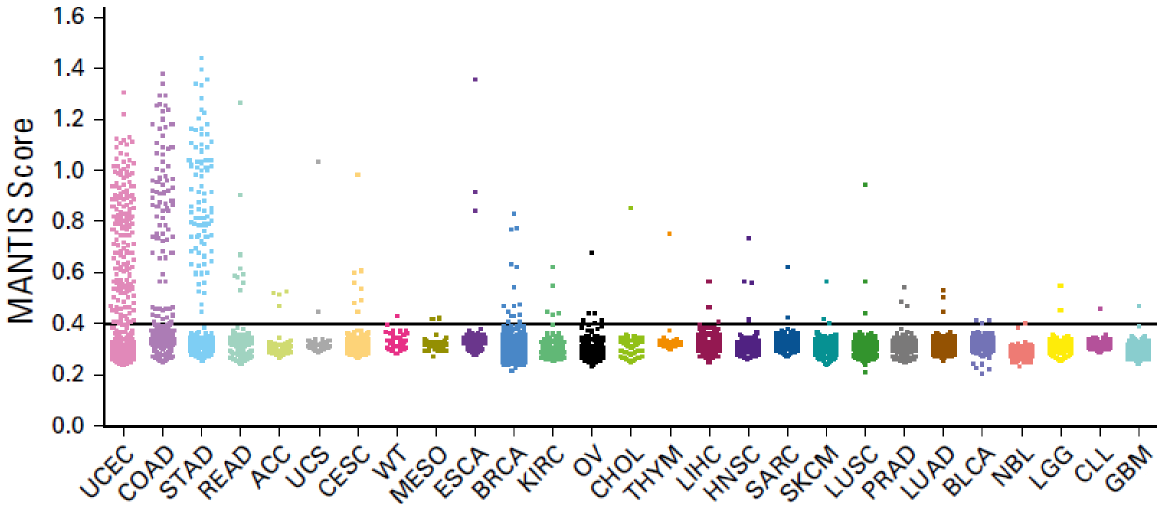

The cancer-specific MSI distribution for the TCGA/TARGET data cohort. The black line at 0.4 represents the MSI-H threshold defined by MANTIS (Kautto et al, Oncotarget 8:7452-7463, 2017). The cancer cohort with any sample of MSI > 0.4 was shown. (figure is referenced to the publication by Bonneville et al, DOI: 10.1200/PO.17.00073 JCO Precision Oncology, published online Oct. 3, 2017).

Marker Description

Microsatellite instability (MSI) correspond to the phenomenon of microsatellite sequence length change caused by base pair insertion or loss in microsatellite regions due to DNA replication errors in the case of deficiency of the MMR system (dMMR status). A large number of studies have shown that MSI is closely associated with tumor development and treatment response. The patients with high levels of MSI (MSI-H) have been shown to be more susceptible to immunotherapies and have improved prognosis.

Mismatch Repair Deficiency (dMMR) Related Genes

Gene	Direction	Mutations	Clinical Significance
**MLH1**	Positive	No	Immunotherapy Sensitive
**MSH2**	Positive	No	Immunotherapy Sensitive
**MSH6**	Positive	No	Immunotherapy Sensitive
**PMS2**	Positive	No	Immunotherapy Sensitive
**MLH3**	Positive	No	Immunotherapy Sensitive

**EPCAM**	Positive	No	Immunotherapy Sensitive
**POLE**	Positive	No	Immunotherapy Sensitive
**POLD1**	Positive	No	Immunotherapy Sensitive
**MDM2**	Negative	No	Immunotherapy Hyperprogressive Disease (HPD)
**MDM4**	Negative	No	Immunotherapy Hyperprogressive Disease (HPD)
**CCND1/FGF3/FGF4/FGF19**	Negative	No	Immunotherapy Hyperprogressive Disease (HPD)
**B2M**	Negative	No	Immunotherapy Resistance
**Clinical significance**	Tumor cells lose the ability to repair DNA replication errors in the case of deficiency of the MMR system (dMMR status), The dMMR usually occur in tumors by somatic mutations or inherited germline mutations in MMR genes. Previous studies have shown that tumors with dMMR status are more likely to respond to immunotherapies than the tumors with proficient MMR.		

3. Mutational Signature (COSMIC V3)

Single base substitutions (SBS96)

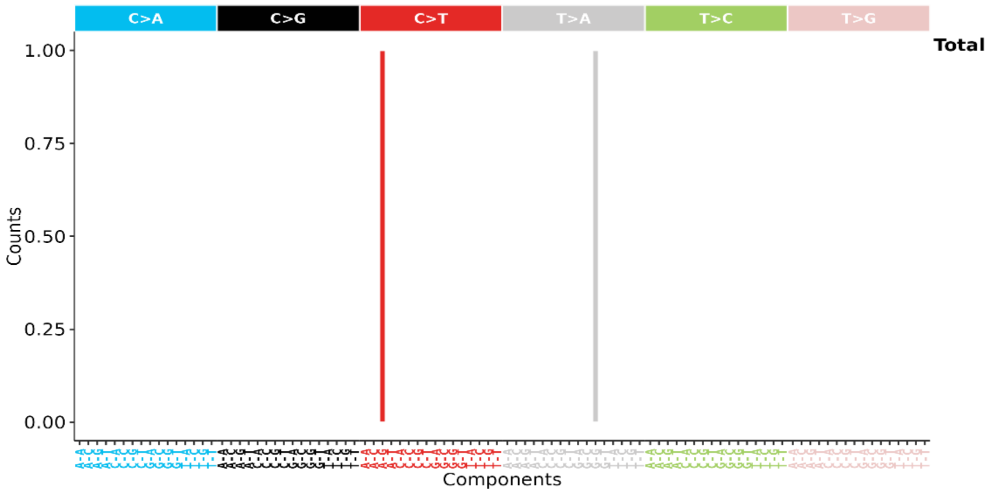

**Description**	SBS signatures result from recurring trinucleotide patterns of the transition/transversion types of somatic single nucleotide variants (SNVs) and their flanking nucleotides. There were substantial differences in the numbers of SBSs between cancer types.

4. Homologous Recombination Repair (HRR)-Related Gene

Gene	Mutation	Gene	Mutation
**BRCA1**	No	**FANCA**	No
**BRCA2**	No	**HDAC2**	No
**PALB2**	No	**ATM**	No
**RAD51**	No	**NBN**	No
**CHEK2**	No	**MRE11A**	No
**POLB**		No	
**Clinical Significance**	Homologous recombination repair (HRR) is a molecular pathway for repair of DNA double-strand breaks. Defects in the genes in this pathway promote genome instability and cancer. However, tumors with deficiency in the HRR pathway are sensitive to the inhibitors of DNA repair enzyme poly-ADP ribose polymerase 1 (PARP1) based on a synthetic lethality mechanism, in that PARP1 is involved in a compensatory DNA repair pathway.		

IV: Therapeutic Interpretations

1. Overview of therapies

Network diagram of gene-disease-therapy relationship	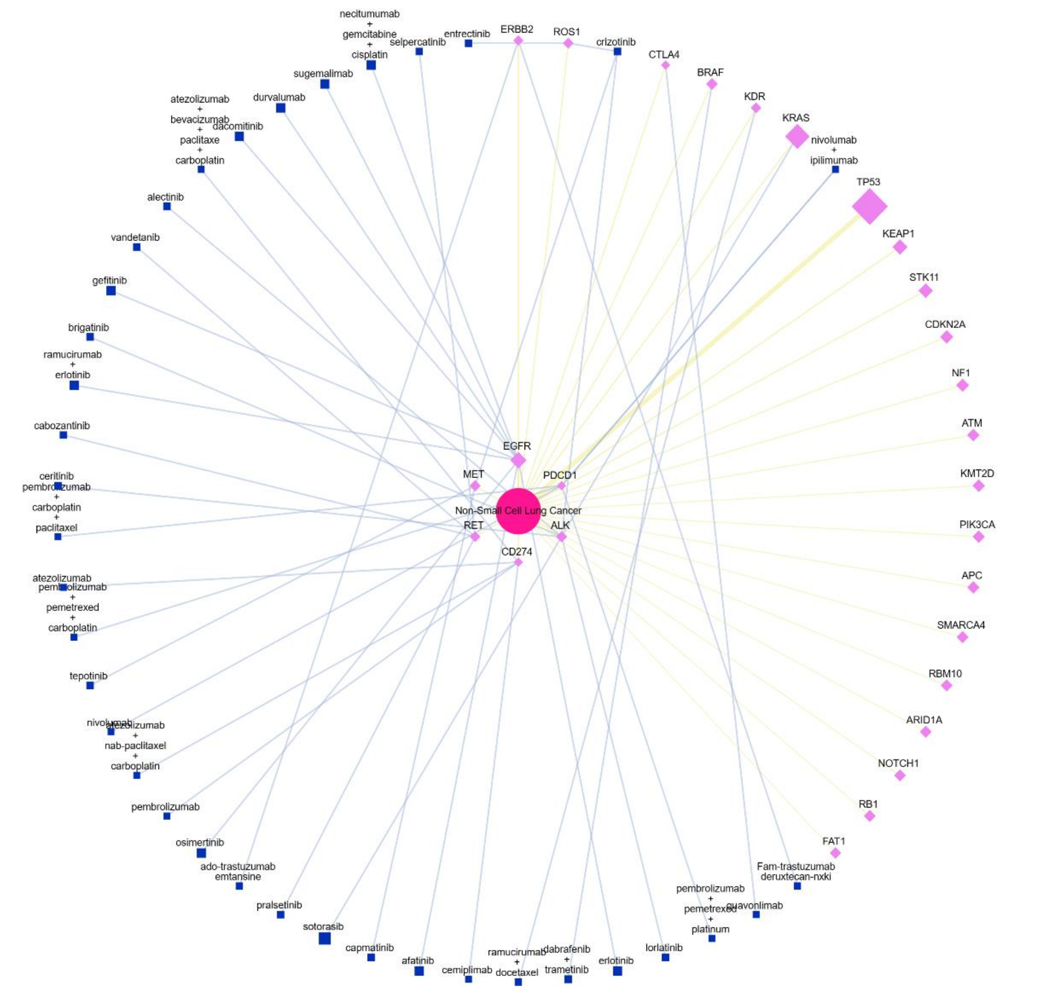
**Description**	The interactive network presentation of the gene-disease-therapy triple-relationship with the cancer as the network center.

2. Targeted Therapy

ALK DB_Match:Fusions	
**Function Interpretations**	
**Gene Function:**	ALK, also known as anaplastic lymphoma kinase encoding protein tyrosine kinase receptors, are closely associated with RTK, belong to the family of insulin receptor. The protein contains an extracellular domain and a hydrophobic monolayer transmembrane intracellular kinase domain (PMID: 9053841), which plays an important role in brain development and affects specific neurons in the nervous system (PMID:9174053, PMID: 11278720). Rearrangement, mutation, or amplification of this gene has been found to lead to a variety of tumors including anaplastic large cell lymphoma, neuroblastoma, and

	non-small cell lung cancer (PMID: 19737948). ALK activates pathways including phospholipase Cγ, JAK, STAT, PI3K-Akt, mTOR, and MAPK signaling cascades that affect cell growth, transformation, and anti-apoptosis (PMID: 12122009).
**Clinical Significance:**	DB_Match: Tri©DB

Therapy Interpretations

**FDA / NCCN for Non-Small Cell Lung Cancer:**	ceritinib
**Evidence Level:**	1
**Indication:**	Ceritinib was approved to be used for treatment of patients with anaplastic lymphoma kinase (ALK)-positive, metastatic non–small cell lung cancer (NSCLC) with disease progression on or intolerance to crizotinib (Xalkori).
**Clinical Trials:**	NCT01828099, NCT01828112, NCT02299505
**Dosage:**	The recommended dose of ceritinib is 750 mg once daily until disease progression or unacceptable toxicity. A recommended dose has not been determined for patients with moderate to severe hepatic impairment. Approximately 60% of patients starting treatment at this dose required at least one dose reduction, and the median time to first dose reduction was 7 weeks. Ceritinib should be discontinued in patients unable to tolerate 300 mg daily.
**Mechanism of Action:**	Ceritinib is a kinase inhibitor. Targets of ceritinib inhibition identified in either biochemical or cellular assays at clinically relevant concentrations include ALK, insulin-like growth factor 1 receptor (IGF-1R), insulin receptor (InsR), and ROS1. Among these, ceritinib is most active against ALK. Ceritinib inhibited autophosphorylation of ALK, ALK-mediated phosphorylation of the downstream signaling protein STAT3, and proliferation of ALK-dependent cancer cells in in vitro and in vivo assays..
**Database source:**	DB_Match: Tri©DB

3. Immunotherapy

PDCD1 DB_Match:Positive Expression

Function Interpretations	
**Gene Function:**	
**Clinical Significance:**	DB_Match:1
**Therapy Summary:**	

Therapy Interpretations	
**FDA / NCCN for Non-Small Cell Lung Cancer:**	pembrolizumab + pemetrexed + platinum
**Evidence Level:**	DB_Match:1
**Indication:**	Pembrolizumab in combination with pemetrexed and platinum was granted regular approval as first-line treatment of patients with metastatic nonsquamous non–small cell lung cancer (NSCLC) with no epidermal growth factor receptor (EGFR) or anaplastic lymphoma kinase (ALK) genomic tumor aberrations.
**Clinical Trials:**	NCT02578680
**Dosage:**	The recommended dose of pembrolizumab in the current indication is 200 mg via intravenous (IV) infusion over 30 minutes every 3 weeks until disease progression, unacceptable toxicity, or up to 24 months in patients without disease progression. Pembrolizumab should be given prior to chemotherapy when given on the same day.
**Mechanism of Action:**	Binding of the PD-1 ligands, PD-L1 and PD-L2, to the PD-1 receptor found on T cells, inhibits T cell proliferation and cytokine production. Upregulation of PD-1 ligands occurs in some tumors and signaling through this pathway can contribute to inhibition of active T-cell immune surveillance of tumors. Pembrolizumab is a monoclonal antibody that binds to the PD-1 receptor and blocks its interaction with PD-L1 and PD-L2, releasing PD-1 pathway-mediated inhibition of the immune response, including the anti-tumor immune response.
**Database source:**	DB_Match: Tri©DB

V: Population Variation Frequency in Diagnostic cancer

**Carrier Rate (GENIE, Top10)**	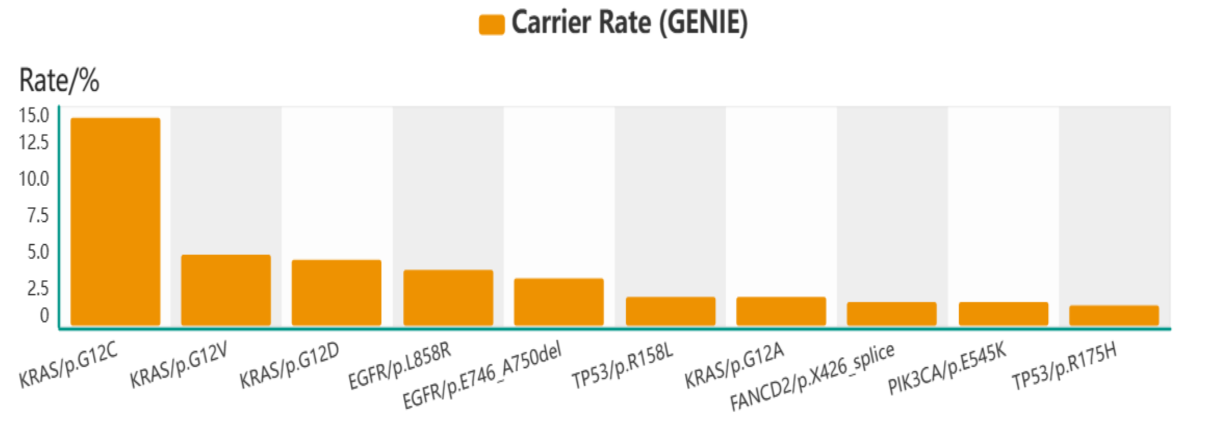
**Carrier Rate (Chinese, Top10)**	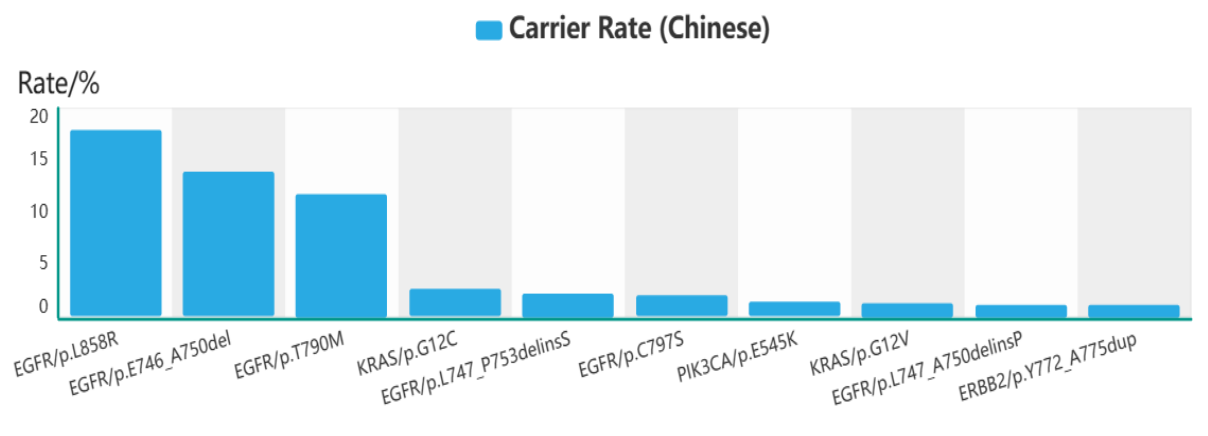
**Carrier Rate (Germline, Top10)**	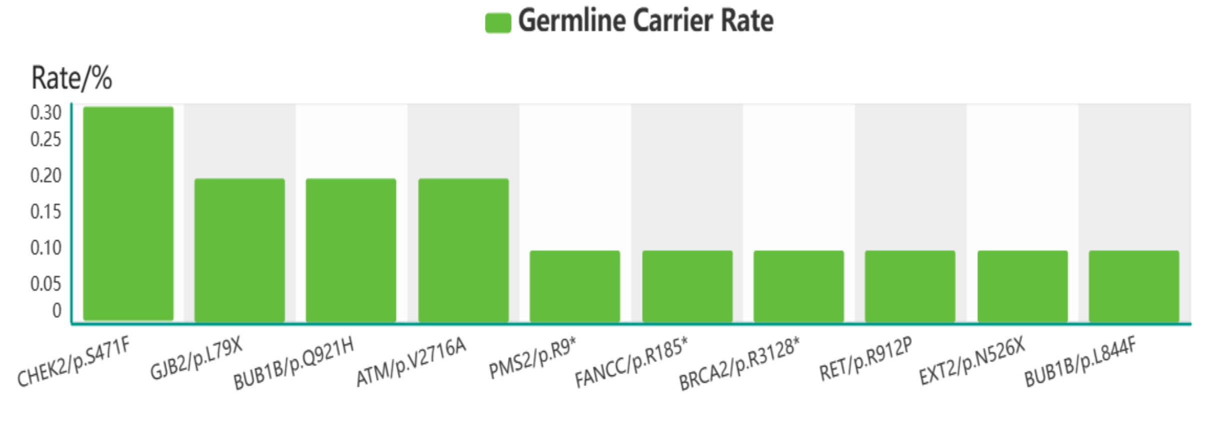
**Description**	The population carrier rate of the variants showed the prevalence of genetic variants in NSCLC in different population settings. The frequency distribution provides an overview of the mutation profile of NSCLC on the level of population.
